# Pressure-induced normal-incommensurate and incommensurate-commensurate phase transitions in CrOCl

**DOI:** 10.1038/srep09647

**Published:** 2015-05-21

**Authors:** Maxim Bykov, Elena Bykova, Leonid Dubrovinsky, Michael Hanfland, Hanns-Peter Liermann, Sander van Smaalen

**Affiliations:** 1 Laboratory of Crystallography, University of Bayreuth, 95440 Bayreuth, Germany; 2 Bavarian Research Institute of Experimental Geochemistry and Geophysics, University of Bayreuth, 95440 Bayreuth, Germany; 3 ESRF, 38043 Grenoble, France; 4 Photon Sciences, DESY, 22607 Hamburg, Germany

## Abstract

The high-pressure behavior of layered CrOCl is shown to be governed by non-bonded interactions between chlorine atoms in relation to a rigid framework composed of Cr and O atoms. The competition between optimizing intra- and interlayer Cl–Cl distances and the general trend towards denser packing defines a novel mechanism for high-pressure phase transitions of inorganic materials. CrOCl possesses an incommensurate phase for 16–51 GPa. Single-crystal x-ray diffraction in a diamond anvil cell provides an accurate description of the evolution of the incommensurate wave with pressure. It thus demonstrates a continuous increase of the amplitude up to 30 GPa, followed by a decrease of the wavelength until a lock-in transition occurs at 51 GPa.

The discovery of high-pressure phases of the elements with unexpectedly complex guest-host and incommensurate structures^1^,^2^,^3^,^4^,^5^ put an end to the myth, that atomic arrangements are simplified under high degrees of compression. For complex compounds systematic studies of incommensurate structures are lacking. Employing single-crystal x-ray diffraction in a diamond anvil cell we show that layered CrOCl^6^ possesses an incommensurate phase for 16–51 GPa and lock-in phases at higher pressures. Detailed and accurate structural studies reveal that the complex behaviour of CrOCl is governed by non-bonded interactions between chlorine atoms, with the incommensurability resulting from the competition between optimizing intra- and interlayer Cl–Cl distances and the general trend towards denser packings. Isostructural FeOCl exhibits the same phase sequence, but with a much narrower pressure range for its incommensurate phase. Similar high-pressure behaviour can be expected for other materials containing different chemical bonds or atomic groups with individual compression behaviours.

In the past decade high-pressure diffraction experiments have uncovered a new type of behaviour of materials at extreme conditions. Contrary to the expectation that on increasing pressure materials should approach high-symmetry close-packed structures, a number of elements possess extraordinary complex incommensurately modulated or self-hosting composite structures at high pressures^1^,^2^,^3^,^4^,^5^. The occurrence of these structures has been rationalized in terms of electronic instabilities^2^,^5^,^7^. Generally, incommensurate structures may appear at high pressures as the result of shifts towards room temperature of the transition temperatures of normal-to-incommensurate phase transitions. This mechanism leads to similar crystal structures for the high-pressure and low-temperature phases, as it has been found in charge-density-wave materials^8^,^9^ and ferroelectrics^10^,^11^. Incommensurability as an intrinsic high-pressure effect is described here for the first time for the compound CrOCl.

Isostructural transition metal oxychlorides *M*OCl (*M* = Ti, V, Cr, Fe) are of particular interest, because they form model systems for studies of the dependence of magnetic properties on the atomic magnetic moments of M^3+^ within a single structure type. Anisotropic magnetic interactions, magnetic frustration and strong magneto-elastic coupling have resulted in a variety of complex magnetically ordered phases at low temperatures^6^,^12^,^13^,^14^,^15^. TiOCl undergoes a phase transition at a pressure of approximately 10–15 GPa towards a twofold superstructure that is reminiscent of the superstructure of the spin-Peierls phase at low temperatures^16^,^17^. A second phase transition takes place at higher pressures^18^, but the powder x-ray diffraction data were of insufficient quality for a structural characterization of the high-pressure phase^17^. Recently, we have developed a methodology of accurate single-crystal x-ray diffraction studies of complex oxychlorides structures at high pressures^19^, and demonstrated its efficiency particularly in an investigation of the fourfold superstructure of FeOCl up to 35 GPa^20^.

We have now measured single-crystal x-ray diffraction of CrOCl up to a pressure of 57 GPa, employing synchrotron radiation at beamline ID09A of the ESRF (on compression) and at beamline P02.2 of PETRA at DESY (on decompression). Analysis of these data has provided an accurate description of the pressure dependence of the crystal structures of CrOCl (Fig. 1). The ambient phase with orthorhombic symmetry *Pmmn* persists up to 13 GPa. A fit of a Vinet equation of state to the volumes of the unit cell resulted in a bulk modulus *K_0_* = 29(2) GPa and its derivative *K_0_'* = 9.5(1). The compressibility is highly anisotropic, with a compression that is much larger in the direction perpendicular to the layers (along **c**) than in directions parallel to the layers (within the **a**, **b** plane; Fig. 2b). This feature can be understood from the crystal structure of MOCl, which is a stacking along the **c**-axis of slabs MOCl that are connected through weak Cl···Cl Van der Waals bonds (Fig. 1a). Bond energies of the latter possess a much more shallow dependence on the interatomic distance than those of chemical bonds M–O and M–Cl, and they are therefore much more compressible (Fig. 3). The anisotropic compressions of CrOCl and FeOCl^20^ are similar up to 13 GPa, which feature supports a common origin for this behavior.

Evidence for a phase transition occurring between 15.3 and 16.4 GPa is provided by Raman scattering (see Supplementary Information) and x-ray diffraction. Diffraction patterns contain superlattice reflections at 16.4 GPa and higher pressures. All Bragg reflections can be indexed on the basis of a unit cell that is closely related to the unit cell of the lattice at ambient conditions, together with a modulation wave vector **q** = (σ_1_, 0, 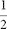). At pressures below 30 GPa, σ_1_ is close to the rational number 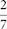 = 0.2857 (Fig. 2c). However, the pressure dependence of the σ_1_ component of the **q**-vector above 30 GPa unambiguously demonstrates the incommensurability of the modulation within this high-pressure phase. The crystal structures were obtained within the superspace approach through refinements against the measured diffraction data at each pressure (see Methods).

The occurrence of a normal-to-incommensurate phase transition can be explained by the collapse of the Van der Waals gap. It was shown for a number of organic and organometallic compounds, that increasing pressure primarily reduces the lengths of the weak intermolecular Van der Waals bonds. However, when these distances reach a lower limit, initially weak interactions approach a strongly repulsive region of the interatomic potential. A further increase of pressure then induces a phase transition, which results in a redistribution of these contacts and thus diminishes the free energy^21^. It was noticed that the lower limit can be estimated as the shortest distance in ambient-pressure structures, e.g. as they can be found in the Cambridge Structural Database (CSD)^22^,^23^. For inter-molecular Cl···Cl contacts, the three shortest distances in the CSD (version November 2013) are 2.961 Å (CEBFEV^24^), 3.027 Å (LENDEP^25^) and 3.028 Å (YUHNIY^26^). Thus, a phase transition may be expected when Cl···Cl distances in MOCl reach a limit of about 3.0 Å. Interlayer Cl···Cl Van der Waals contacts become the shortest Cl···Cl distances above ~11 GPa (Fig. 3). At 12.95 GPa (the highest pressure below the phase transition at which diffraction was measured) this distance is 3.069 Å, close to the limit of 3.0 Å. The observation of a phase transition at 15.9 ± 0.6 GPa thus is in complete agreement with the behaviour of molecular crystals. It is demonstrated below, that the incommensurate crystal structure allows for a further compression of the compound without the need to shorten the Cl···Cl contacts significantly. Average interlayer Cl···Cl distances are nearly equal at equal pressures for CrOCl and FeOCl^20^ (Supplementary Table 7), while both compounds exhibit phase transitions at nearly equal pressures. These observations reinforce the conclusion of a common origin for the high-pressure behavior of these compounds.

Within the MOCl structure type, the intralayer Cl···Cl distance along **a** is determined by the geometry of the poorly compressible M–O framework at the center of each slab. So, at pressures, where the interlayer Cl···Cl distance reaches the limit of 3 Å, the intralayer Cl···Cl distance along **a** is, with 3.8 Å, still larger than twice Van der Waals radius of Cl (Fig. 3). On the local scale (taking into account only one pair of neighboring slabs), this space between Cl atoms can be used in two different ways to achieve a denser packing of Cl atoms. In the first model, the slabs bulge towards each other (region **A** in Fig. 4), resulting in an increase of the gap between Cl atoms neighboring along **a**, thus allowing Cl atoms of neighboring slabs to interpenetrate (Fig. 1 b–d), resulting in a denser packing. In the second model, slabs bend inward (region **B** in Fig. 4), resulting in a reduction of the space between Cl atoms neighboring along **a**. However, interlayer Cl···Cl distances become longer, thus allowing layers to close in, again resulting in a denser packing.

Because the M–O framework is covered by Cl atoms at both sides, the appearance of region **A** on one side will immediately lead to region **B** on the other side of the slab (Fig. 4). This results in doubling of the unit cell along the **c** axis which explains the rational component 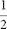 of the modulation wave vector. The accurate structure models reveal that the incommensurate crystal structures represent an antiphase buckling of adjacent slabs indeed (Fig. 1 b–d). This is represented by in-phase *z*-displacements of M, O and Cl atoms belonging to the same slab and possessing the same *x*-coordinates in the average structure (see *t*-plots in Supplementary Fig. 4). The structure models furthermore show that the major part of the modulation is given by displacements of Cl atoms in the direction of **a**, providing densifications of the packing in addition to the effects of buckling of the layers. The superspace symmetry of the incommensurate structure does not allow for atomic displacements along **b**. This is in agreement with the suggested mechanism of the phase transition, because the Cl···Cl distances along **b** are much smaller than twice the Van der Waals radius of Cl atom, leaving no room for further decrease. Displacements of Cl atoms are severely restricted by the rigidity of the M–O and M–Cl bonds. They prevent a single most favorable Cl···Cl distance to be achieved for all Cl atoms, thus explaining the incommensurability and the modulations of varying wavelengths as a function of pressure.

The discovery of the incommensurate phase in CrOCl has triggered us to re-investigate the high-pressure phase diagram of FeOCl in the vicinity of 15 GPa. Indeed, incommensurate satellite reflections have now been found in the diffraction pattern of FeOCl at 15.0 GPa with **q** = (0.260, 0, 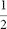), while the **q**-vector is commensurate with σ_1_ = 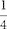 at the next measured pressure of 22.7 GPa. The incommensurate phase of FeOCl exists in a range of pressures of at most a few GPa wide. Different radii of Fe^3+^ and Cr^3+^ lead to different M–O distances, and therefore to a different packings of Cl atoms, explaining the different periods of the modulations in FeOCl and CrOCl. The slightly lower transition pressure in FeOCl can be explained by the smaller cation size of Cr^3+^ than Fe^3+^, which makes the CrOCl slabs more rigid. Therefore, more energy (higher pressure) is required to distort the Cr–O framework than to distort the Fe–O framework. Rigorous assessment of the impact of slight differences in ambient-pressure structures, different cation sizes and different electronic configurations on the observed differences between the **q**-vectors and their pressure dependence would require additional experiments on isostructural compounds.

While the range of incommensurability of FeOCl is small, the intermediate structure of CrOCl persists up to 51 GPa. Compression from 16.4 to 30.3 GPa is accompanied by a gradual increase of modulation amplitudes. At 30.3 GPa the amplitudes saturate and further compression enhances the σ_1_ component of the **q**-vector. Apparently, the initial growth of modulation amplitudes is required to avoid shortening of interlayer Cl···Cl distances with diminishing of the interlayer spacing. An upper bound is reached, when unfavorably short intralayer Cl···Cl distances would occur. At higher pressures the evolution of the structure follows the expected behaviour of buckled slabs: increase of the σ_1_ component of the **q**-vector allows to reduce the *a*-axis avoiding the energy-demanding large contraction of Cr–O distances.

At 57.2 GPa (highest pressure achieved) two commensurate phases are found to coexist in CrOCl. This indicates that two phase transitions take place between 51 and 57.2 GPa. One is a lock-in transition where σ_1_ jumps to 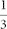 and a sixfold, 3*a* × *b* × 2*c* superstructure is formed. The second commensurate high-pressure phase is a threefold superstructure (see Supplementary Information). The transition is not accompanied by significant changes in molar volume. Therefore, no changes in connectivity (e.g. appearance of interlayer bonding) are expected. We could not obtain direct evidence of the exact sequence of phase transitions between 51 and 57.2 GPa. However, the smaller unit cell of the threefold superstructure may suggest that it is developed at higher pressures than the sixfold superstructure of the lock-in phase. On decompression, the lock-in phase is preserved down to at least 47.5 GPa, with the commensurate-to-incommensurate transformation taking place between 47.5 and 40.5 GPa. These observations provide evidence for strong first-order character of the lock-in transition. Furthermore, it supports the idea that the threefold superstructure is the stable phase at the highest pressures.

We have shown that high-pressure behaviour of MOCl is governed by strongly non-bonded inter- and intralayer Cl···Cl contacts. In the vicinity of 15 GPa interlayer Cl···Cl contacts reach the strongly repulsive region of the interatomic potential at ~3 Å interatomic separation. This results in a phase transition towards an incommensurately modulated phase by which shorter Cl···Cl distances are avoided. MOCl are not molecular compounds, where generally there are more degrees of freedom regarding the variation of molecular arrangement. Nevertheless, there are voids within the structure, which allow Cl displacements along **a**. The rigid framework of M–O bonds, in turn, implies restrictions on the positions of Cl atoms. Therefore, a single most favorable configuration cannot be achieved. In CrOCl the high-pressure structure is incommensurate up to at least 51 GPa. Between 51 and 57 GPa a lock-in phase transition to a sixfold 3*a* × *b* × 2*c* superstructure takes place, while at still higher pressures a threefold superstructure is formed. Apparently, there is a tendency for the simplification of the structure towards higher pressures. The importance of non-bonded interactions described here for the high-pressure behavior of CrOCl will have implications for the understanding of high-pressure phase diagrams of other classes of inorganic materials, but it may also bear relevance for the stress properties of Van der Waals-bonded heterostructures^27^.

## Methods

### Sample preparation

Single crystals of CrOCl were prepared by the gas transport technique using CrCl_3_ (99.9%, Alfa) and Cr_2_O_3_ (99.997%, Alfa) as starting reagents, and using HgCl_2_ as transport reagent^6^. Single crystals of FeOCl were prepared by gas transport from a stoichiometric mixture of FeCl_3_ (99.99%) and Fe_2_O_3_ (99.999%)^15^. The high-quality single crystals of CrOCl and FeOCl along with small ruby spheres were placed inside diamond anvil cells equipped with Boehler-Almax diamonds (culet size 250 μm). Ne was used as a pressure-transmitting medium.

### X-ray diffraction

Single-crystal x-ray diffraction experiments were performed at the beamlines ID09A (ESRF, Grenoble, France) and P02.2 (PetraIII, Hamburg, Gemany) using monochromatic X-ray radiation of wavelengths 0.414 and 0.290 Å respectively. The diffraction data were collected in a sequence of 1° oscillations over a total scan range of 80° around the vertical (ω) axis. Diffracted intensities were collected with Mar555 flat panel and PerkinElmer XRD 1621 area detectors.

### Data analysis

Lattice parameters, components of the **q**-vector and integrated intensities were obtained from the measured images using the computer program *CrysAlisPro*^28^. Bragg reflections overlapping with reflections from the diamonds or the pressure-transmitting medium were excluded from the data integration procedure. Outliers were removed (usually 1–2 per data set) according to procedures recently implemented in Jana2006^29^.

### Crystal structures

Crystal structures were obtained within the superspace approach using the superspace group *Pmmn*(σ_1_, 0, 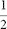)00*s*. Structure refinements of the atomic coordinates and modulation amplitudes were performed with the software Jana2006^29^. They resulted in a good fit to the diffraction data at each pressure. Further details on structure models are given in the supplementary information.

## Author Contributions

S.v.S. planned and coordinated the study. M.B., E.B., L.D., M.H. and H.-P.L. performed the diffraction experiments. M.B. analysed the data. M.B., L.D. and S.v.S. wrote the paper.

## Additional Information

**How to cite this article**: Bykov, M. *et al.* Pressure-induced normal-incommensurate and incommensurate-commensurate phase transitions in CrOCl. *Sci. Rep.* 5, 9647; DOI:10.1038/srep09647 (2015).

## Supplementary Material

Supplementary Information Supplementary information

## Figures and Tables

**Figure 1:**
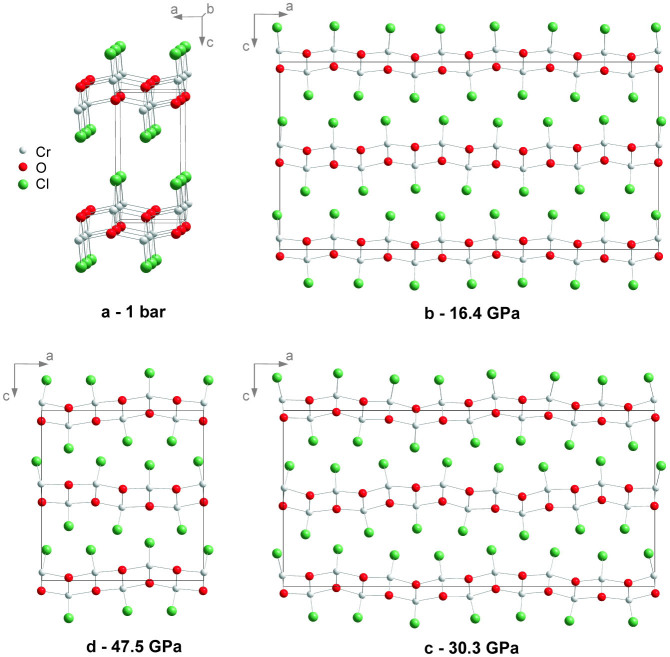
Crystal structures of CrOCl at different pressures. (a) Perspective view along the **b**-axis of the ambient-pressure crystal structure. (b) Approximate 7*a* × *b* × 2*c* superstructure at 16.4 GPa. (c) Approximate 7*a* × *b* × 2*c* superstructure at 30.3 GPa. (d) 3*a* × *b* × 2*c* superstructure at 47.5 GPa as obtained on decompression.

**Figure 2:**
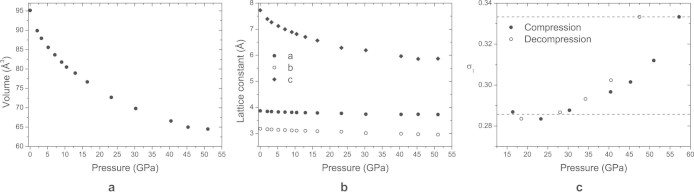
Pressure dependencies of CrOCl of (a) the unit-cell volume; (b) the lattice parameters; and (c) the σ_1_ component of the q-vector. Dashed lines indicate rational values 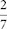 and 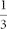.

**Figure 3:**
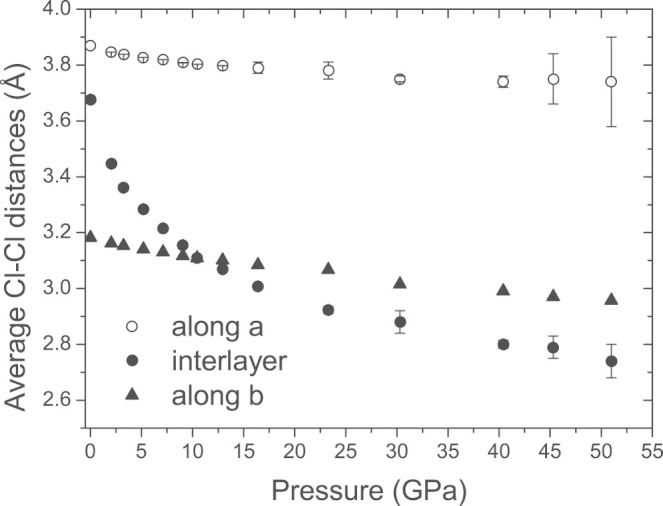
Pressure dependence of the Cl···Cl distances in CrOCl. For pressures above 15 GPa the averaged distance is given, for averaging over the phase *t* of the modulation. If error bars are not shown, they are smaller than the size of the symbol.

**Figure 4:**
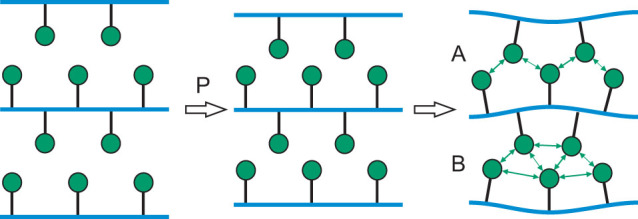
Schematic representation of regions in the high-pressure phase of MOCl. Double arrows represent Cl···Cl contacts that have achieved more favorable distances due to the modulation.
