# Supplementary material for: Pressure-induced normal-incommensurate and incommensurate-commensurate phase transitions in CrOCl
Source: Sci Rep. 2015 May 21;5:9647. doi: 10.1038/srep09647 (PMC4440982; doi:10.1038/srep09647)
Supplement: Supplementary Information [file srep09647-s1.pdf]

Pressure-induced normal–incommensurate and incommensurate–commensurate phase transitions in CrOCl

Supplementary information

Maxim Bykov[1], Elena Bykova[1,2], Leonid Dubrovinsky[2], Michael Hanfland[3], Hanns-Peter Liermann[4] and Sander van Smaalen[1]*

[1]Laboratory of Crystallography, University of Bayreuth, 95440 Bayreuth, Germany,

[2]Bavarian Research Institute of Experimental Geochemistry and Geophysics, University of Bayreuth, 95440 Bayreuth, Germany,

[3]ESRF, 38043 Grenoble, France

[4]Photon Science, DESY, 22607 Hamburg, Germany.

*e-mail: smash@uni-bayreuth.de

Contents:

1. Experiments and data processing.

2. Details of structure solution and refinement.

3. Pressure dependence of the Raman scattering of CrOCl.

4. Selected *t*-plots of the incommensurately modulated structure of CrOCl at 23.3 GPa.

5. Tables of selected interatomic distances and angles.

1. Experiments and data processing

Single-crystal X-ray diffraction experiments have been performed on crystals loaded in BX90 diamond anvil cells (DACs)[1] equipped with Boehler-Almax diamonds[2]. Pressures were obtained from the shifts of the R_1 fluorescence line of ruby[3]. The fluorescence was measured directly before and immediately after the data collections, in order to establish the magnitudes of pressure variation during each experiment. Neon has been used as pressure-transmitting medium. It crystallizes at approximately 4.8 GPa[4], but its diffraction peaks became clearly visible only at 13 GPa. Therefore, for higher pressures, the lattice parameter of Ne could be used as an additional pressure indicator[5]. The maximum difference between pressures measured before and measured after the data collections, and determined by different methods did not exceed 0.7 GPa.

Integrated intensities of Bragg reflections were obtained from the measured diffraction images by the software *CrysAlisPro*[6]. It appeared necessary to convert the format of the Mar555 images to Mar345 format. Custom-made software available at beamline ID09A was used for this purpose. Perkin Elmer images in .tif format were converted to the esperanto image type[6] that is supported by the latest version of *CrysAlisPro*[6]. Components of the modulation wave vector were refined simultaneously with the orientation matrix against the observed positions of the reflections, using the computer program *NADA*[7] as implemented in *CrysAlisPro*. Outliers were removed according to procedures recently implemented in JANA2006[8]. Equations of state were determined with the software EosFit7c[9] applied to the measured lattice parameters and unit-cell volumes.

For Raman spectroscopy experiments, a BX90 DAC with standard design diamond anvils (250 μm culet size) was used. Raman spectra were measured in backscattering geometry, employing a Dilor XY Raman spectrometer using an Ar^+ ion laser (Coherent Innova 300) with a wavelength of 514.5 nm, and possessing a spectral resolution of 1 cm^{-1}. The laser power was kept below 2 mW, in order to avoid laser-heating of the sample. Phonon frequencies were obtained by fitting Pearson VII functions to the experimental peaks.

2. Details of structure solution and refinement

Structure refinements for the low-pressure phase of CrOCl

CrOCl keeps its ambient-pressure structure up to 14.5 GPa. Crystal structures at these pressures were successfully refined against each of the eight data sets of X-ray diffraction data measured at pressures between 0.0001 and 12.95 GPa (Supplementary Table 1). The structure published by Forsberg[10] was used as starting model. Due to the limited coverage of reciprocal space in the high-

pressure diffraction experiments at the ESRF, it appeared necessary to use a smaller number of independent parameters in the refinements than the three coordinates and nine atomic displacement parameters (ADPs) allowed by symmetry. It was chosen to use isotropic ADPs for Cr and O, thus reducing the number of ADP parameters from 9 to 5. The Cl atoms can be expected to possess large and anisotropic displacement amplitudes, because they form the boundary of the Van der Waals gap. Therefore, Cl was given anisotropic ADPs. This model leads to a pronounced drop in *R*-factors and is supported by a Hamilton test (0.005 significance level), while anisotropic refinement of Cr and O atoms does not lead to a significant lowering of the agreement factors. Data obtained upon decompression contained more reflections, which allowed refinement anisotropic ADPs for all atoms (Supplementary Table 2).

Larger mosaic spreads at higher pressures reduced the importance of extinction. Therefore, an extinction correction was not applied at pressures above 7.1 GPa. All refinements smoothly converged to excellent fits to the diffraction data (Supplementary Tables 1 and 2).

Structure refinement of the incommensurate high-pressure phase of CrOCl

CrOCl is found to undergo a phase transition at a pressure between 15.3 and 16.4 GPa. The phase transition is evidenced by the presence of weak superlattice reflections in the diffraction patterns at 16.4 GPa and higher pressures. All observed Bragg reflections could be indexed on the basis of a unit cell that is closely related to the unit cell below 14.5 GPa, together with a modulation wave vector $\mathbf{q} = (\sigma_1\ 0\ \frac{1}{2})$. Analysis of the diffraction symmetry and the reflection conditions revealed two possible superspace groups: $Pmmn(\sigma_1\ 0\ \frac{1}{2})00s$ and $Pmmn(\sigma_1\ 0\ \frac{1}{2})000$. These groups are different settings of the superspace group $Pmnm(\sigma_1\ 0\ \frac{1}{2})000$ (No. 59.1.10.6)[11] and can be obtained from each other by a translation of the origin by $(00\frac{1}{2}0)$ in superspace. The superspace group $Pmmn(\sigma_1\ 0\ \frac{1}{2})00s$ has been chosen in order to keep the atomic coordinates in the basic structure close to those in low-pressure phase structure model.

Atomic modulation functions were described by truncated Fourier series for all the atoms. Up to second-order harmonics were used at all pressures except 16.4 GPa, where second-order satellites were not observed, and therefore only first-order harmonics could be used. The ambient-pressure structure model was used as a starting model for the refinements of the basic structure against the main reflections. Subsequently, small but arbitrary values were given to the modulation amplitudes. Refinement of all parameters against all reflections resulted in a smooth convergence and a good fit to the diffraction data at each pressure (Supplementary Tables 1 and 2).

At pressures below 30 GPa, σ_1 is close to the rational number $\frac{2}{7} = 0.2857$. This value would imply a commensurate modulation that can alternatively be described as a superstructure with a 14-fold, $7a \times b \times 2c$ supercell. The superspace group of the modulated-structure description implies different symmetries of the supercell in dependence on the section t_0 of superspace. Possible 3-dimensional (3D) space groups are *Pcmn* for $t_0 = 0 + \frac{n}{14}$, *Pmmn* for $t_0 = \frac{1}{28} + \frac{n}{14}$ ($n = 0,1,\cdots,13$), and $P2_1mn$ for other values of t_0. In addition to the refinement of the incommensurate structure model, superstructure models were tested by commensurate superspace refinements with t_0 equal to 0, $\frac{1}{28}$ and 0.050508, respectively. Differences between the *R*-factors of these four refinements did not exceed 0.01% (Supplementary Table 3). As a consequence, it is impossible to distinguish between different superstructures and between incommensurate and commensurate modulations, solely on the basis of the refinements. This ambiguity may be the result of having available only highly incomplete data sets. Furthermore, the 14-fold superstructure would allow satellites up to the seventh order, but only first- and second-order satellites were observed, which again diminishes the sensitivity of the diffraction to a possible commensurability of the modulation.

On the other hand, the pressure dependence of the σ_1 reveals an incommensurate modulation for pressures between 30 and 51 GPa, while phase transitions between 16.4 and 51 GPa were not observed. This strongly suggests the incommensurability of this high-pressure phase of CrOCl. However, a commensurate-to-incommensurate transition at approximately 30 GPa cannot be entirely excluded on the basis of the present data.

Structure refinement of the incommensurate high-pressure phase of FeOCl at 15 GPa

In FeOCl at 15 GPa satellite reflections can be indexed with the **q**-vector $(0.26, 0, \frac{1}{2})$. The superspace symmetry and the starting structure model for the refinement are the same as for CrOCl. Atomic modulation functions were described as first-order harmonics. Refinement of all parameters against all reflections resulted in a smooth convergence and a good fit to the diffraction data (Supplementary Table 4).

Structure refinement of the commensurate high-pressure phases of CrOCl

At P = 57.2 GPa the diffraction pattern contains two sets of satellite reflections. One set can be indexed as a lock-in phase of the incommensurately modulated phase at lower pressures, employing $\sigma_1 = \frac{1}{3}$. The second set can be indexed with the different modulation wave vector $\mathbf{q}^2 = (\frac{1}{3}, 0, \frac{1}{3})$. On decompression, at P = 47.5 GPa only the lock-in phase survived. Therefore, the two sets of satellite reflections were treated as originating in different parts of the crystal. Unfortunately, we were not able to obtain acceptable fits to the diffraction data at 57.2 GPa. We

ascribe this problem to the limited scattering information contained in the data sets, together with the fact that main reflections of the two phases overlap with each other. Consequently, we discuss only main structural features of the second phase, while bond distances, angles and displacement parameters are not reliable. For the structure solution and refinement, the second phase was treated as having monoclinic symmetry (space group $P2_1/m$) with a = 6.910, b = 2.8999, c = 9.325 Å, β = 95.86° (Supplementary Table 5). The cell transformation $\mathbf{a} + \mathbf{c}, -\mathbf{b}, 2\mathbf{a} - \mathbf{c}$ leads to a $3a \times b \times 3c$ X-centered monoclinic supercell with centering vectors $(\frac{1}{3}, 0, \frac{1}{3})$ and $(\frac{2}{3}, 0, \frac{2}{3})$. On decompression, at P = 47.5 GPa only the lock-in phase survived. It was described by the same superspace group and the same basic structure as the incommensurately modulated structure at lower pressures. Refinements of the commensurately modulated structure converged smoothly to a good fit to the diffraction data (Supplementary Table 2). The best fit to the diffraction data was obtained for the section $t_0 = \frac{1}{12}$ of superspace (Supplementary Table 6). This structure model corresponds to a superstructure with a sixfold, $3a \times b \times 2c$ supercell with space group *Pmmn*.

Supplementary Table 1: Experimental details on compression of CrOCl at ID09A (ESRF).

	0.0001 GPa	2.05 GPa	3.25 GPa	5.20 GPa
**Crystal data**				
a (Å)	3.8683(1)	3.8461(1)	3.8382(1)	3.8272(1)
b (Å)	3.1823(4)	3.1618(1)	3.1530(1)	3.1403(1)
c (Å)	7.726(8)	7.3924(17)	7.2652(19)	7.1203(19)
V ($Å^3$)	95.14(7)	89.90(2)	87.92(2)	85.58(2)
**Data collection**				
No. of reflections:				
measured,	164	196	190	182
independent,	51	62	63	60
observed (I > 3 σ(I))	51	59	61	52
R_{int} (obs/all)	0.0441/0.0441	0.0288/0.0288	0.0310/0.0310	0.0322/0.0323
Redundancy	3.216	3.161	3.016	3.033
$(\sin\theta/\lambda)_{max}$($Å^{-1}$)	0.764	0.783	0.785	0.787
**Refinement**				
No. of parameters	10	10	10	10
R_F(obs)/wR_F (all)	0.0423/0.0569	0.0272/0.0438	0.0242/0.0390	0.0286/0.0374
$\Delta\rho_{min}/\Delta\rho_{max}$($e\cdot Å^{-3}$)	-0.57/0.59	-0.36/0.27	-0.36/0.39	-0.42/0.50

	7.10 GPa	9.05 GPa	10.45 GPa	12.95 GPa
**Crystal data**				
a (Å)	3.8190(1)	3.8083(1)	3.8024(1)	3.7978(2)
b (Å)	3.1299(1)	3.1170(1)	3.1088(1)	3.1009(2)
c (Å)	6.9996(17)	6.889(2)	6.811(2)	6.703(6)
V ($Å^3$)	83.67(2)	81.78(2)	80.51(2)	78.94(7)
**Data collection**				
No. of reflections:				
measured,	166	192	188	158
independent,	58	61	61	56
observed (I > 3 σ(I))	56	46	50	47
R_{int} (obs/all)	0.0361/0.0361	0.0523/0.0525	0.0437/0.0438	0.0250/0.0250
Redundancy	2.862	3.148	3.082	2.821
$(\sin\theta/\lambda)_{max}$($Å^{-1}$)	0.789	0.787	0.729	0.732
**Refinement**				
No. of parameters	10	9	9	9
R_F(obs)/wR_F (all)	0.0344/0.0552	0.0327/0.0418	0.0395/0.0459	0.0425/0.0619
$\Delta\rho_{min}/\Delta\rho_{max}$($e\cdot Å^{-3}$)	-0.33/0.46	-0.74/0.74	-0.5/1.0	-0.55/1.04

	16.4 GPa	23.3 GPa	30.3 GPa	40.4GPa
**Crystal data**				
a (Å)	3.7866(3)	3.7698(5)	3.7406(12)	3.7323(6)
b (Å)	3.0848(3)	3.0669(5)	3.0156(18)	2.9902(6)
c (Å)	6.564(6)	6.286(8)	6.19(3)	5.967(7)
V ($Å^3$)	76.67(7)	72.68(11)	69.8(3)	66.59(8)
**q**-vector	(0.2869(5),0, ½)	(0.2835(3),0, ½)	(0.2877(6),0, ½)	(0.2967(4),0, ½)
**Data collection**				
No. of reflections:				
measured (main/sat1/sat2)*	165/393/0	140/331/253	145/358/305	136/304/238
independent (main/sat1/sat2),	55/106/0	44/96/91	48/101/94	43/90/86
observed (I > 3 σ(I)) (main/sat1/sat2)	46/63/0	39/65/50	43/61/49	36/54/44
R_{int} (obs/all)	0.0519/0.0523	0.0665/0.0674	0.0560/0.0577	0.0558/0.0573
Redundancy	3.466	3.134	3.325	3.178
$(\sin\theta/\lambda)_{max}$($Å^{-1}$)	0.778	0.784	0.790	0.762
**Refinement**				
No. of parameters	17	23	23	23
R_F (obs) (all/main)	0.0420/0.0400	0.0408/0.0369	0.0604/0.0529	0.0582/0.0436
R_F (obs) (sat1/sat2)[1]	0.0486/ –	0.0445/0.0431	0.0638/0.0754	0.0670/0.0855
wR_F (all) (all/main)	0.0541/0.0524	0.0552/0.0455	0.0734/0.0637	0.0686/0.0493
wR_F (all) (sat1/sat2)	0.0612/ –	0.0617/0.0771	0.0785/0.1119	0.0855/0.1267
$\Delta\rho_{min}/\Delta\rho_{max}$($e\cdot Å^{-3}$)	-0.71/0.75	-0.57/0.51	-0.99/0.79	-0.86/0.87

Continued on the next page…

Supplementary Table 1. Continued from the previous page

	45.3 GPa	51.0 GPa
**Crystal data**		
a (Å)	3.7348(11)	3.726(3)
b (Å)	2.9702(18)	2.9574(20)
c (Å)	5.86(3)	5.87(5)
V ($Å^3$)	65.0(3)	64.5
**q**-vector	(0.3015(5),0, ½)	(0.3120(4),0, ½)
**Data collection**		
No. of reflections:		
measured (main/sat1/sat2)	136/338/287	134/276/256
independent (main/sat1/sat2),	49/92/89	38/81/81
observed (I > 3 σ(I)) (main/sat1/sat2)	39/46/39	34/50/44
R_{int} (obs/all)	0.0573/0.0593	0.0458/0.0467
Redundancy	3.309	3.330
$(\sin\theta/\lambda)_{max}$($Å^{-1}$)	0.774	0.792
**Refinement**		
No. of parameters	23	23
R_F (obs) (all/main)	0.0575/0.0534	0.0504/0.0373
R_F (obs) (sat1/sat2)	0.0528/0.0845	0.0496/0.1022
wR_F (all) (all/main)	0.0723/0.0637	0.0563/0.0402
wR_F (all) (sat1/sat2)	0.0766/0.1334	0.0606/0.1467
$\Delta\rho_{min}/\Delta\rho_{max}$($e\cdot Å^{-3}$)	-0.85/1.07	-1.01/0.81

*Here and in the following tables notation “sat *n*” correspond to n^{th}-order satellites.

Supplementary Table 2: Experimental details on decompression of CrOCl at P02.2 (DESY).

	47.5 GPa	40.5 GPa	28.0 GPa	18.5 GPa
**Crystal data**				
a (Å)	3.707(3)	3.7404(8)	3.772(2)	3.8127(8)
b (Å)	2.943(3)	3.0133(8)	3.050(2)	3.1086(9)
c (Å)	5.91(4)	5.93(1)	6.17(3)	6.379(11)
V ($Å^3$)	64.5(4)	66.88(12)	71.0(4)	75.61(14)
**q**-vector	(1/3 ,0, ½)	(0.3022(3),0, ½)	(0.2867(2),0, ½)	(0.2836(2),0, ½)
**Data collection**				
No. of reflections:				
measured (main/sat1/sat2)	296/640/603	291/653/613	277/655/639	312/743/704
independent (main/sat1/sat2),	74/145/144	78/153/149	77/159/161	90/171/170
observed ($I > 3\ \sigma(I)$) (main/sat1/sat2)	70/85/86	72/94/87	67/97/81	76/92/52
R_{int} (obs/all)	0.0463/0.0514	0.0417/0.0467	0.0340/0.0423	0.0386/0.0522
Redundancy	4.240	4.097	3.957	4.081
$(\sin \theta/\lambda)_{max}$($Å^{-1}$)	0.986	0.977	0.950	0.963
**Refinement**				
No. of parameters	23	23	23	23
R_F (obs) (all/main)	0.0280/0.0213	0.0263/0.0242	0.0306/0.0245	0.0224/0.0179
R_F (obs) (sat1/sat2)	0.0296/0.0414	0.0234/0.0365	0.0356/0.0384	0.0237/0.0424
wR_F (all) (all/main)	0.0403/0.0235	0.0360/0.0268	0.0410/0.0260	0.0406/0.0215
wR_F (all) (sat1/sat2)	0.0503/0.0735	0.0349/0.0717	0.0553/0.0701	0.0521/0.1208
$\Delta\rho_{min}/\Delta\rho_{max}$($e \cdot Å^{-3}$)	-0.71/0.73	-0.63/0.49	-0.63/0.58	-0.54/0.51

	14.5 GPa	5.5 GPa	0.0001 GPa
**Crystal data**			
a (Å)	3.8123(13)	3.8268(2)	3.8601(3)
b (Å)	3.1109(11)	3.1429(2)	3.1771(3)
c (Å)	6.65(2)	7.212(3)	7.708(5)
V ($Å^3$)	78.9(3)	86.74(4)	94.53(6)
**q**-vector	–	–	–
**Data collection**			
No. of reflections:			
measured (main/sat1/sat2)	359/–/–	436/–/–	456/–/–
independent (main/sat1/sat2),	94/–/–	85/–/–	91/–/–
observed ($I > 3\ \sigma(I)$) (main/sat1/sat2)	64/–/–	74/–/–	78/–/–
R_{int} (obs/all)	0.0266/0.0308	0.0260/0.0272	0.0675/0.0691
Redundancy	3.819	5.129	5.011
$(\sin \theta/\lambda)_{max}$($Å^{-1}$)	0.971	0.953	0.940
**Refinement**			
No. of parameters	14	14	14
R_F (obs) (all/main)	0.0263/0.0263	0.0165/0.0165	0.0302/0.0302
R_F (obs) (sat1/sat2)	–/–	–/–	–/–
wR_F (all) (all/main)	0.0287/0.0287	0.0209/0.0209	0.0370/0.0370
wR_F (all) (sat1/sat2)	–/–	–/–	–/–
$\Delta\rho_{min}/\Delta\rho_{max}$($e \cdot Å^{-3}$)	-0.47/0.50	-0.37/0.39	-0.62/0.73

Supplementary Table 3: Comparison of the refinements of the high-pressure phase of CrOCl between 16.4 and 30.3 GPa using different models.*

	16.4 GPa	23.3 GPa	30.3 GPa	28.0 GPa	18.5 GPa
Incommensurate:					
R_F (all)	0.0420	0.0481	0.0605	0.0306	0.0224
R_F (main)	0.0400	0.0411	0.0529	0.0245	0.0179
R_F (sat1)	0.0485	0.0532	0.0641	0.0356	0.0237
R_F (sat2)		0.0649	0.0751	0.0384	0.0424
Commensurate:					
$t_0 = 0$					
R_F (all)	0.0420	0.0481	0.0606	0.0306	0.0223
R_F (main)	0.0400	0.0411	0.0527	0.0245	0.0172
R_F (sat1)	0.0487	0.0532	0.0643	0.0357	0.0237
R_F (sat2)	–	0.0648	0.0762	0.0384	0.0422
Commensurate:					
$t_0 = 1/28$					
R_F (all)	0.0420	0.0481	0.0606	0.0305	0.0223
R_F (main)	0.0400	0.0411	0.0527	0.0245	0.0172
R_F (sat1)	0.0487	0.0531	0.0642	0.0357	0.0237
R_F (sat2)	–	0.0649	0.0762	0.0383	0.0422
Commensurate:					
$t_0 = 0.050508$					
R_F (all)	0.0420	0.0481	0.0606	0.0305	0.0223
R_F (main)	0.0400	0.0411	0.0527	0.0245	0.0172
R_F (sat1)	0.0487	0.0531	0.0643	0.0357	0.0237
R_F (sat2)	–	0.0649	0.0762	0.0383	0.0422

*Reflections were averaged according to point symmetry 2*mm* for all refinements

Supplementary Table 4: Experimental details on structure refinement of FeOCl at 15.0 GPa.

**Crystal data**	
a (Å)	3.6615(11)
b (Å)	3.1937(11)
c (Å)	6.780(13)
V (Å^3)	79.29(16)
**q**-vector	(0.261 ,0, ½)
**Data collection**	
No. of reflections:	
measured (main/sat)	252/503
independent (main/sat)	61/121
observed (I > 3σ(I)) (main/sat)	50/52
Redundancy	4.148
$(\sin \theta/\lambda)_{max}$(Å^{-1})	0.719
R_{int}(obs/all)	0.0571/0.0615
**Refinement**	
No. of parameters	21
R_F (obs) (all/main/sat)	0.0550/0.0549/0.0555
wR_F (all) (all/main/sat)	0.0677/0.0626/0.0906
$\Delta\rho_{min}/\Delta\rho_{max}$(e·Å^{-3})	0.85/-1.16

Supplementary Table 5: Crystal data on CrOCl high-pressure phases observed at 57.2 GPa.

	Lock-in Phase (3*a*×*b*×2*c*)	Phase 2 (3*a*×*b*×3*c*)
a (Å)	3.6788(13)	6.910(16)
b (Å)	2.8999(9)	2.8950(9)
c (Å)	5.812(18)	9.325(9)
β (°)	90	95.86(18)
V ($Å^3$)	62.0(2)	185.6(5)
**q**-vector	(1/3 ,0, ½)	–
Space group	*Pmmn*	$P2_1/m$
**Data collection**		
No. of reflections:		
measured (main/sat1/sat2/sat3)[3]	288/807/744/412	1131/–/–/–
independent (main/sat1/sat2/sat3)	94/199/194/104	538/–/–/–
observed (I > 3σ(I)) (main/sat1/sat2/sat3)	65/68/65/9	247/–/–
Redundancy	3.814	2.102
$(\sin \theta/\lambda)_{max}$($Å^{-1}$)	1.086	1.093
R_{int}(obs/all)	22.17/26.98	17.96/19.85
**Refinement**		
No. of parameters	23	28
R_F (obs) (all/main)	0.0882/0.0905	0.0807/0.0807
R_F (obs) (sat1/sat2/sat3)	0.0955/0.0711/0.0891	–/–/–
wR_F (all) (all/main)	0.1333/0.0.1114	0.1153/0.1153
wR_F (all) (sat1/sat2/sat3)	0.1397/0.1463/0.3552	–/–/–
$\Delta\rho_{min}/\Delta\rho_{max}$(e·$Å^{-3}$)	-2.79/3.21	-1.95/1.83

Supplementary Table 6: Comparison of the refinements of the high-pressure lock-in phase of CrOCl at 47.5 GPa on decompression using different models.*

	$t_0 = 0$	$t_0 = 1/12$	t_0 = general	Incommensurate
Supercell space group	*Pcmn*	*Pmmn*	$P2_1mn$	
R_{all} (I > 3σ(I))	0.0981	0.0279	0.0344	0.0491
R_{main} (I > 3σ(I))	0.0316	0.0212	0.0216	0.0260
R_{sat1} (I > 3σ(I))	0.0889	0.0297	0.0322	0.0461
R_{sat2} (I > 3σ(I))	0.2658	0.0414	0.0674	0.1071
wR_{all}	0.1191	0.0402	0.0467	0.0610
wR_{main}	0.0337	0.0233	0.0234	0.0278
wR_{sat1}	0.1117	0.0503	0.0532	0.0645
wR_{sat2}	0.2984	0.0734	0.0979	0.1370

* Reflections were averaged according to point symmetry 2*mm* for all refinements

3. Pressure dependence of the Raman scattering of CrOCl

Raman spectra of CrOCl were recorded at 13 pressures within the range 5–31 GPa (Supplementary Fig. 1). The full representation of the vibrational modes of CrOCl in space group *Pmmn* is:

$$\Gamma_{tot} = 3A_g + 2B_{1u} + 3B_{2g} + 2B_{2u} + 3B_{3g} + 2B_{3u},$$

where A_g, B_{2g} and B_{3g} are Raman active. At low pressures three strong Raman active modes are clearly observed (Supplementary Fig. 1). They can be identified with Ag modes according to Fausti et al[3]. The three additional weakly active modes may have either B2g or B_{3g} symmetry. All Raman peaks gradually shift to higher energies with pressure (Supplementary Fig. 2). Dramatic changes in Raman spectrum are observed in the pressure region between 15.3 and 17.3 GPa, thus manifesting a phase transition.

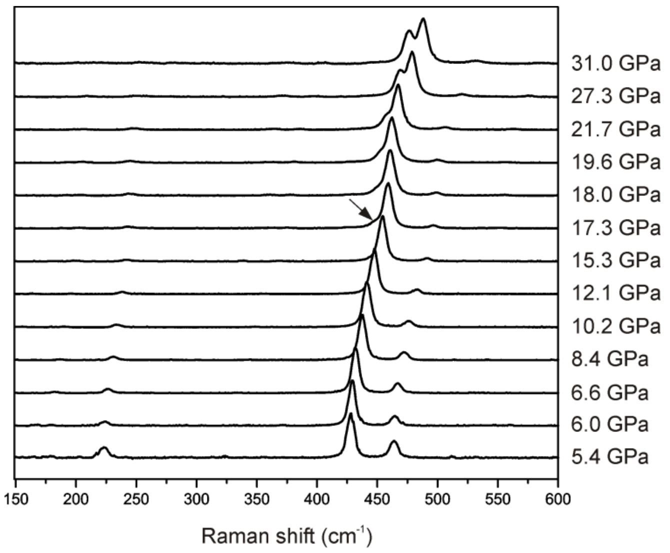

Supplementary Figure 1. Raman spectra of CrOCl at different pressures. The arrow points at the strongest additional peak appearing in the course of the phase transition.

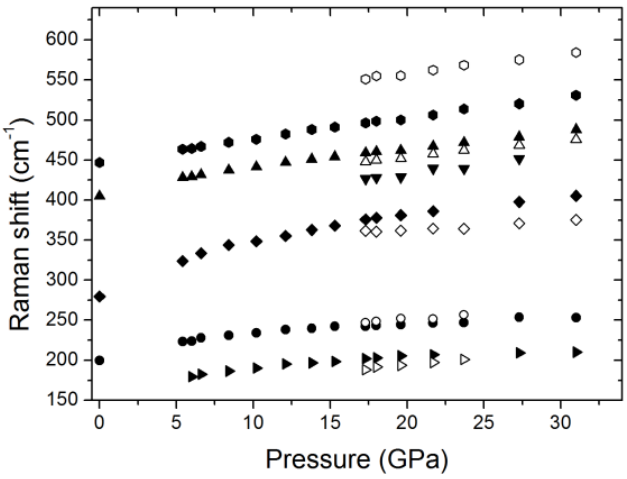

Supplementary Figure 2. Variation of the Raman shift frequencies with applied pressure.

4. *t*-plots of the incommensurately modulated structure of CrOCl at 23 GPa

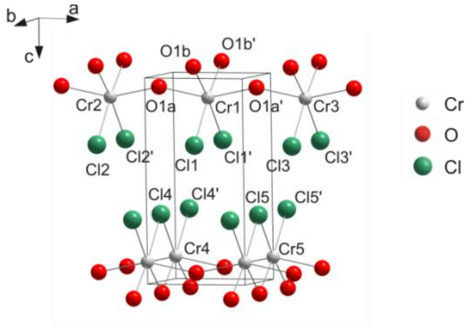

Supplementary Figure 3. Average structure of CrOCl. The atomic labeling is shown as it has been used in the Supplementary Figure 4 and in the Supplementary Table 8.

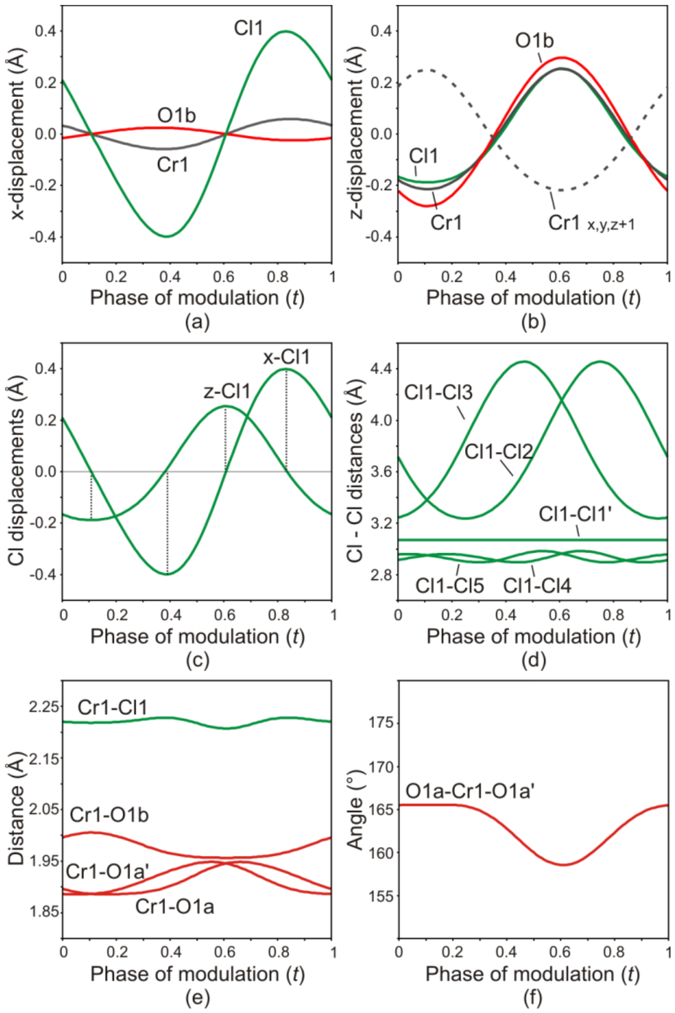

Supplementary Figure 4. Selected *t*-plots of the incommensurately modulated structure of CrOCl at 23.3 GPa. (a) *x*-displacements of Cr, O and Cl atoms. (b) *z*-displacements of Cr, O, Cl atoms. (c) *x*- and *z*-displacements of the Cl atom; vertical dashed lines are drawn at *t* values corresponding to maximal and minimal displacements. (d) Cl···Cl distances. (e) Cr-O and Cr-Cl distances. (f) O-Cr-O angle.

5. Tables of interatomic distances and angles

Supplementary Table 7. Average interlayer Cl···Cl distances (Å) in CrOCl and in FeOCl.

Pressure (GPa)	FeOCl	CrOCl
0.0001	3.680(6)*	3.678(8)
15.0	3.06(3)	
16.4		3.007(9)
22.7	2.92(3)**	
23.3		2.924(8)

* Data from [12]

** Data from [13]

Supplementary Table 8: Selected interatomic distances and angles. For pressures above 15 GPa distances and angles have been averaged over the phase *t* of the modulation.

Pressure (GPa)	Cr–O1a (Å)	Cr1–O1b (Å)	Cr1–Cl1 (Å)	Cl1···Cl2 (Å)	Cl1···Cl1' (Å)
0.0001	1.976(4)	2.036(12)	2.316(7)	3.8683(1)	3.1823(4)
2.05	1.969(3)	2.012(8)	2.301(5)	3.8461(1)	3.1618(1)
3.25	1.956(2)	2.023(8)	2.302(4)	3.8382(1)	3.1530(1)
5.20	1.955(3)	2.009(10)	2.281(5)	3.8272(1)	3.1403(1)
7.10	1.946(3)	2.013(9)	2.274(4)	3.8190(1)	3.1299(1)
9.05	1.941(3)	2.009(11)	2.258(5)	3.8083(1)	3.1170(1)
10.45	1.934(3)	2.002(12)	2.262(6)	3.8024(1)	3.1088(1)
12.95	1.933(5)	1.988(15)	2.246(7)	3.7978(2)	3.1009(2)
16.4	1.921(12)	1.993(11)	2.241(6)	3.79(2)	3.0848(3)
23.3	1.912(5)	1.976(15)	2.220(8)	3.78(3)	3.0669(5)
30.3	1.90(5)	1.95(2)	2.194(12)	3.75(1)	3.0156(18)
40.4	1.886(15)	1.95(2)	2.173(13)	3.74(2)	2.9902(6)
45.3	1.89(5)	1.94(3)	2.140(15)	3.75(9)	2.9702(18)
51.0	1.88(8)	1.95(2)	2.164(11)	3.74(16)	2.9574(20)

	Cl1···Cl4 (Å)	O1a–Cr–O1a' (Å)	Cl1–Cr1–Cl1' (Å)	O1b–Cr1–O1b' (Å)	O1b–Cr1–Cl1(Å)
0.0001	3.676(8)	156.7(8)	86.8(2)	102.8(8)	85.2(4)
2.05	3.446(6)	155.3(6)	86.8(2)	103.6(6)	84.8(3)
3.25	3.360(5)	157.6(5)	86.46(16)	102.4(4)	85.6(3)
5.20	3.284(6)	156.4(6)	87.00(19)	102.8(7)	85.1(3)
7.10	3.216(5)	157.8(6)	86.99(16)	102.1(6)	85.5(3)
9.05	3.156(6)	157.5(7)	87.3(2)	101.8(7)	85.5(4)
10.45	3.110(6)	158.8(6)	86.8(2)	101.9(8)	85.6(4)
12.95	3.069(7)	158.5(10)	87.3(3)	102.5(8)	85.1(6)
16.4	3.007(9)	161.2(9)	87.0(2)	101.4(7)	85.8(5)
23.3	2.923(12)	163.0(11)	87.4(3)	101.8(8)	85.3(6)
30.3	2.88(4)	162.2(16)	86.8(5)	101.6(12)	85.6(9)
40.4	2.800(14)	166.5(17)	86.9(5)	100.0(12)	86.3(9)
45.3	2.79(4)	167.1(19)	87.9(6)	100.0(15)	85.8(11)
51.0	2.74(6)	170.5(15)	86.2(6)	98.5(12)	87.4(10)

References:

1. Kantor, I. *et al.* BX90: a new diamond anvil cell design for X-ray diffraction and optical measurements. *Rev. Sci. Instrum.* **83,** 125102 (2012).

2. Boehler, R. & De Hantsetters, K. New anvil designs in diamond-cells. *High Press. Res.* **24,** 391–396 (2004).

3. Mao, H. K., Xu, J. & Bell, P. M. Calibration of the ruby pressure gauge to 800 kbar under quasi-hydrostatic conditions. *J. Geophys. Res.* **91,** 4673–4676 (1986).

4. Klotz, S., Chervin, J.-C., Munsch, P. & Le Marchand, G. Hydrostatic limits of 11 pressure transmitting media. *J. Phys. D. Appl. Phys.* **42,** 075413 (2009).

5. Fei, Y. *et al.* High-Pressure Geoscience Special Feature: Toward an internally consistent pressure scale. *Proc. Natl. Acad. Sci.* **104,** 9182–9186 (2007).

6. Rothkirch, A. *et al.* Single-crystal diffraction at the Extreme Conditions beamline P02.2: procedure for collecting and analyzing high-pressure single-crystal data. *J. Synchrotron Radiat.* **20,** 711–720 (2013).

7. Schönleber, A., Meyer, M. & Chapuis, G. NADA – a computer program for the simultaneous refinement of orientation matrix and modulation vector(s). *J. Appl. Crystallogr.* **34,** 777–779 (2001).

8. Friese, K. & Grzechnik, A. Refinement of high pressure single-crystal diffraction data using Jana2006. *High Press. Res.* **33,** 196–201 (2013).

9. Angel, R. J., Alvaro, M. & Gonzalez-Platas, J. EosFit7c and a Fortran module (library) for equation of state calculations. *Z. Kristallogr.* **229,** 405–419 (2014).

10. Forsberg, H.-E. On the Structure of CrOCl. *Acta Chem. Scand.* **16,** 777 (1962).

11. Stokes, H. T., Campbell, B. J. & van Smaalen, S. Generation of (3 + d)-dimensional superspace groups for describing the symmetry of modulated crystalline structures. *Acta Crystallogr. A.* **67,** 45–55 (2011).

12. Lind, M. D. Refinement of the crystal structure of iron oxychloride. *Acta Crystallogr. B.* **26,** 1058–1062 (1970).

13. Bykov, M. *et al.* High-pressure behavior of FeOCl. *Phys. Rev. B* **88,** 014110 (2013).
